# Effects of Tai Chi Yunshou exercise on community-based stroke patients: a cluster randomized controlled trial

**DOI:** 10.1186/s11556-018-0206-x

**Published:** 2018-12-12

**Authors:** Guanli Xie, Ting Rao, Lili Lin, Zhengkun Lin, Tianshen Xiao, Ming’ge Yang, Ying Xu, Jinmei Fan, Shufang Lin, Jinsong Wu, Xiaodong Feng, Li Li, Jing Tao, Lidian Chen

**Affiliations:** 1 College of Rehabilitation Medicine, Fujian University of Traditional Chinese Medicine, 1 Huatuo Road, Minhou Shangjie, Fuzhou, 350122 Fujian China; 2 Affiliated Rehabilitation Hospital, Fujian University of Traditional Chinese Medicine, Fuzhou, China; 3 Traditional Chinese Medicine Rehabilitation Research Center of State Administration of Traditional Chinese Medicine, Fuzhou, People’s Republic of China; 4 Rehabilitation medical technology Joint National Local Engineering Research Center, Fuzhou, China; 5 Fujian Collaborative Innovation Center for Rehabilitation Technology, Fuzhou, China; 6 The First Affiliated Hospital of Henan University of Traditional Chinese Medicine, Zhengzhou, China; 7 The Second Affiliated Hospital of Shandong University of Traditional Chinese Medicine, Jinan, China

**Keywords:** Tai chi Yunshou exercises, Wave hands in the cloud, Balance dysfunction, Stroke, Community-dwelling, Cluster randomized controlled trial

## Abstract

**Backgroud:**

Tai Chi Chuan was used for stroke survivors with balance impairments. However, even a short-form of Tai Chi Chuan includes forms that make the exercise challenging for the stroke survivors. Tai Chi Yunshou (wave hands in the cloud) is the “mother” form and the fundamental form of all Tai Chi Chuan styles, which is considered more suitable and feasible for stroke survivors with balance impairments. So this study was designed to evaluate the effects of Tai Chi Yunshou exercise on community-based stroke patients with balance dysfunctions.

**Methods:**

A total of 250 participants from 10 community health centers (5 per arm) were selected and randomly allocated into Tai Chi Yunshou exercise group (TC group) or a balance rehabilitation training group (control group) in an equal ratio. Participants in the TC group were received Tai Chi Yunshou exercise training five times per week for 12 weeks and those in control group were received balance rehabilitation training five times per week for 12 weeks. Outcome assessments including Berg Balance Scale (BBS), Time up to go test (TUGT), Modified Barthel Index (MBI) were measured at baseline, 4 weeks, 8 weeks, 12 weeks and followed-up 6 weeks (18 weeks), 12 weeks (24 weeks). Intention-to-treat analysis was performed. Analysis of variance of repeated measures was used to assess between-group differences.

**Results:**

A total of 244 participants, 120 in the TC group and 124 in the rehabilitation group, were included in final analysis. There was no siginificant difference in Tai Chi Yunshou and balance rehabilitation training on the improvement of balance ability and mobility (*P* = 0.531 and *P* = 0.839, respectively) after adjustment for baseline. However, there was significant difference between two groups on improvement of motor funtion (*P* = 0.022), fear of falling (*P* < 0.001) and depression (*P* = 0.035) for the post stroke patients. No adverse events were reported during the study.

**Conclusion:**

Tai Chi Yunshou and balance rehabilitation training led to improved balance ability and functional mobility, and both are suitable community-based programs that may benefit for stroke recovery and community reintegration. Our data demonstrated that a 12-week Tai Chi Yunshou intervention was more effective in motor function, fear of falling and depression than balance rehabilitation training. Future studies examining the effectiveness of Tai Chi Yunahou as a balance ability improvement strategy for community-dwelling survivors of stroke are recommended.

**Trial registration:**

Chinese Clinical Trail Registry: ChiCRT-TRC-13003641. Registration date: 22 August, 2013.

## Background

Stroke has become the second most common cause of death and the third most common cause of disability worldwide [1]. Stroke alters people’s lives by affecting their motor function, mobility, cognitive function and more, leading to restrictions in their basic activities of daily living (ADL) [2]. Of all the dysfunctions caused by stroke, balance impairment is a common cause of disability and has the greatest impact on ADL performance [3–5], which directly affects the quality of life for patients and their outcomes [6]. Therefore, interventions that could alleviate balance impairments in this target population were suggested to improve their balance and functional mobility [7].

Although many balance programs including standing balance practices [8], and motor relearning programs [9] have been used for the acute and subacute phases of stroke patients, community-dwelling stroke survivors have fewer options due to real and perceived barriers [10]. Moreover, one-on-one sessions are needed for chronic stroke patients [11], which are not easy to obtain in community health centers (CHCs), and supervision by health care professionals is required. These factors all increase the expenditure related to stroke rehabilitation, which becomes a main economic burden for families [12]. So a lower-cost, effective, easily prescribed intervention for stroke survivors with balance dysfunctions is needed in the community [13].

A growing body of evidence has demonstrated that Tai Chi Chuan exercise can help to improve or maintain balance ability, motor function, mobility, fear of falling and quality of life in the elderly [14, 15]. The safety of Tai Chi Chuan has also been demonstrated among chronic patients including stroke survivors [16–21]. As a mind-body exercise, Tai Chi Chuan can induce relaxation and tranquility of the mind, improve balance function and reduce the risk of falling by enhancing the proprioceptive sensibility which was impaired in stroke survivors [22–25]. In addition, once they have mastered the Tai Chi Chuan forms, stroke survivors can continue to exercise Tai Chi Chuan anywhere, anytime without limitation to guidance or supervision of a profession. Increasing the ability of stroke survivors to perform their own rehabilitation regime anywhere, anytime should also be an important goal for rehabilitation [17]. Tai Chi Chuan was recommend to use in community for stroke patients [16, 17, 26].

There are multitudinous styles of Tai Chi Chuan, such as Yang-style, Sun-style, and Chen-style, each having short and long forms (form refers to number of movements). The movements vary from style to style. In the current researches, the Yang-style (24-form) [16, 18], Sun-style (12-form) [17], Simplified Tai Chi Chuan (6-form) [27] were used to explore the effects of Tai Chi Chuan on the balance of stroke survivors. The results indicated that Tai Chi Chuan is a safe, community-based exercise program [16, 26], and can improve the balance [28], reduce the fall rate, improve the quality of life [18, 28] for this target population. However, even a short-form of Tai Chi Chuan (such as 6-form) included forms (e.g., part the wild horse’s mane on both sides, lower the body and stand on one leg) that make the exercise challenging for the stroke survivors, especially those with balance dysfunctions [10, 29]. Tai Chi Yunshou movement (wave hands in the cloud), known as the “mother form” and the fundamental form of all styles of Tai Chi Chuan, fully embodies the basic pricinple of Tai Chi Chuan [30]. During movement, shoulders are balanced and relaxed, and body weight is evenly distributed on the soles of the feet, the body remains in an upright position with the shoulders aligned over the hips, the waist is the center or axis and directs the movement of the arms, legs, and eyes, the head, trunk, and pelvis rotate as a single unit, aligned over a stable base in the feet [30]. We believe that Tai Chi Yunshou movement maybe suitable and feasible for stroke survivors with balance impairments. The hypothesis that Tai Chi Yunshou movement can improve the balance dysfunction for the community-based stroke survivors was also considered. Thus, a cluster randomized controlled study was designed to evaluate the multi-dimensional effects of a 12-week Tai Chi Yunshou exercise program on physical health and mental health for a group of community-dwelling stroke survivors.

## Methods

### Participants

A single-blind cluster randomized, parallel-controlled trial was conducted. Participants were recruited from CHCs run by three research centers (The Rehabilitation Hospital affiliated to Fujian University of Traditional Chinese Medicine, Fuzhou, China; The First Affiliated Hospital of Henan University of TCM Zhengzhou, China; and The Second Affiliated Hospital of Shandong University of Traditional Chinese Medicine, Jinan, China) via newspaper publicity, posting up posters, sending leaflets and referrals from neurologists and physical therapists. Ethical approvals were granted by ethics committees in all research centers. All participants were fully informed about the protocol and signed the written informed consent form prior to participation.

Inclusion criteria included the following: (1) Aged 45 to 75 years; (2) Diagnosed with stroke according to the criteria adopted by the Fourth National Cerebral Vascular Disease Conference [31] and confirmed by CT or MRI; (3) First onset of stroke more than 3 months prior; (4) Balance dysfunctions caused by stroke rather than other encephalopathies; (5) Ability to walk more than 6 m independently or assisted; (6) Agreement from patient or his/her legal guardian and signed written informed consent; and (7) Mini-Mental State Exam score > 24 and ability to understand, receive guidance and implement the exercises. The exclusion criteria included the following: (1) Existing diseases affecting training; (2) Impaired vestibular function; (3) Severe visual or hearing impairments that affected the training; (4) Sensory aphasia; (5) Prior Tai Chi Chuan experience in the last 6 months; (6) Serious complications after stroke; (7) Serious medical conditions such as severe heart disease, cancer, or gastrointestinal hemorrhage; and (8) Participation in other clinical trials that affected the results of this study.

### Sample size

The sample size for the study was estimated based on our preliminary experiment, which suggested an intervention group would have a mean increase of 8.5 points (SD = 2.8) on the BBS. We expected that 25 eligible cases would be enrolled in each cluster (m = 25) based on the results of a pre-epidemiological investigation. With a type I error of 5 and 90% power, along with a 0.05 correlation coefficient intra-CHC, a sample size of 114 was required according to the formula [32]:

$$ n=\frac{2\left[{\left({\mu}_{\alpha }+{\mu}_{\beta}\right)}^2{\sigma}^2\right]}{\sigma^2}\left[1+\left(m-1\right)\rho \right] $$

To allow for a 10% dropout rate, 125 participants were required for each group.

### Randomization and blinding

To prevent cross-contamination, residents of each CHC were defined as a cluster. CHCs were randomly assigned to either the TC group or the control group in a 1:1 ratio via the PLAN algorithm in the statistical software SAS version 8.2 (SAS Institute, Inc., Cary, NC). The CHCs randomization program was safe-guarded by a specified project manager who did not participate in any other processes. Although it was impossible to blind the exercise coaches and subjects in this study, the coaches did not participant in any assessment of outcomes. The screeners, outcome evaluators and statisticians were blind throughout the study. Meanwhile, all outcome assessors and the data analyst did not participate in screening or assigning.

### Intervention

The participants were allowed to maintain routine medical therapy (such as the management of the blood pressure, blood glucose, and accompanying symptoms et al.) depending on personal situation and visit their primary care physicians during the study period. Both groups received health education via a bulletin board, pamphlets and lectures. Both programs were carried out in the CHCs. All subjects completed a 12-week intervention.

Tai Chi Yunshou exercise occurred five times per week for 60 min each session. The form originated from the 24 short-form Tai Chi Chuan exercise normed by General Administration of Sports of China [33]. To exercise wave hands in the cloud, one must stand straight, move arms and legs with the waist at the axis, and breathe in a relaxed manner. The details of Tai Chi Yunshou exercise was reported at protocol of this study [34]. Each session comprised 45 min of exercise plus a 15-min warm-up and cool-down. Exercise was directed by five qualified coaches with more than 5 years of experience in physical education. The coach can adjust the length of the training according to patients’ personal situation, and allocate properly. Individuals who can not complete the exercise continuously could be allowed to finish intermittently. But each session should be no less than 15 min. Meanwhile, another supervisor who was trained and qualified by research team was responsible for recording the training of individuals to ensure the quality of training for each community.

Balance rehabilitation training was also carried out five times per week for 60 min each session. The training form originated from the ‘Technical Specification of Common Rehabilitation Therapy’ (2012) published by the Chinese Association of Rehabilitation Medicine [35]. The balance rehabilitation training includes static balance training, dynamic balance training, bobath training, walking training and so on according to the patient’s functional level and condition.

### Follow-up period

All subjects completed a 12 weeks of follow-up conducted by telephone weekly and home visits monthly. During follow-up period, no participants were asked to receive additional intervention.

### Outcome measures

Demographic characteristics were obtained from an interviewer administered questionnaire at recruiting. All outcomes measures were assessed at baseline and at the end of week 4, week 8, and week 12 of the intervention as well as at 6-week follow-up (week 18) and 12-week follow-up (week 24). The design of this study has been reported previously [34].

The Chinese version of Berg Balance Scale (BBS), with good reliability and validity in Chinese stroke patients [36], was used to measure balance function as the primary outcome. The total of 14 items were used to measure static and dynamic aspects of balance function on a scale from 0 to 4 for each item [36]. Higher scores indicate better balance function.

Static balance was measured by Single Leg Stance Test (SLST) [37]. Individuals were tested with eyes open; they were asked to stand on either their left or right leg and were instructed to keep their legs from touching and to maintain single-leg stance for as long as possible. The duration of standing was record. The best performance was recorded for each leg after three tests. A score was recorded as 0 s if the individual could not do it. Fugl-Meyer Assessment (FMA) was used to measure motor function, with 33 items that reflect upper-limb motor function and 17 items that reflect lower-limb motor function on a scale from 0 to 2 for each item [38]. Mobility was measured by Timed-Up-and-Go-Test (TUGT) [39]. The subjects were asked to rise from an armless chair, walk 3 m with orthosis or assistive device as required, turn round, and return to the seat as fast as possible. A sign was placed at the 3-m point, and a research assistant blinded to the subject allocation recorded three times of a subject to complete the task via a stop-watch and calculated the average according to previous study [28, 37]. ADL was measured by simplified Chinese version of Modified Barthel Index (MBI), with good reliability and validity in Chinese population, with the total 10 items [40]. Fear of falling was measured by Chinese version of Modified Falls Efficacy Scale (MFES), with good reliability and validity in Chinese population [41]. This scale asked participants to perceive their efficacy at avoiding falls during several relatively nonhazardous activities of daily living. The Medical Outcomes, Chinese version SF-36 including physical component summary (PCS) and mental component summary (MCS) parts, with good reliability and validity in Chinese population [42], was used to measure the quality of life, with higher scores predicting better perceived quality of life. Depression was measured by Beck Depression Inventory (BDI), a self-rating scale widely used in the assessment of depressive symptoms [43]. The BDI contains 21 groups of statements that describe mental conditions that are scored from 0 to 3 for each item. Higher scores indicate more severe depression [43].

### Safety evaluation

Any adverse events that occurred were recorded on a case report form. The adverse events were defined as any unfortunate medical events incident during the study period (such as cardiovascular events, cerebral vascular events and falls).

### Statistical analysis

All statistical analyses were performed using IBM SPSS for Windows (version 20.0). A two-sided *P* value ≤0.05 was considered to be statistically significant. The outcomes were analyzed on an intention-to-treat (ITT) basis. The missing data were imputed by using the Fully Conditional Specification (FCS) algorithm of multiple imputations, which is an iterative Markov Chain Monte Carlo (MCMC) method (random number: 20171030). All continuous variables are described using means and standard deviations or median and inter-quartile range. T-tests or Mann-Whitney tests were used for continuous variables and Pearson’s chi-squared or Fisher’s exact tests were used for categorical variables to compare differences between groups. The repeated measures analysis of varianc was used to measured between-group differences. The variables of FMA, MBI, MEFS, BDI, which was different between groups at baseline, were treated as a covariate. The level of significance was 0.05 being the Bonferroni-adjusted alpha for across-time comparisons within the groups.

## Results

**Fig. 1:**
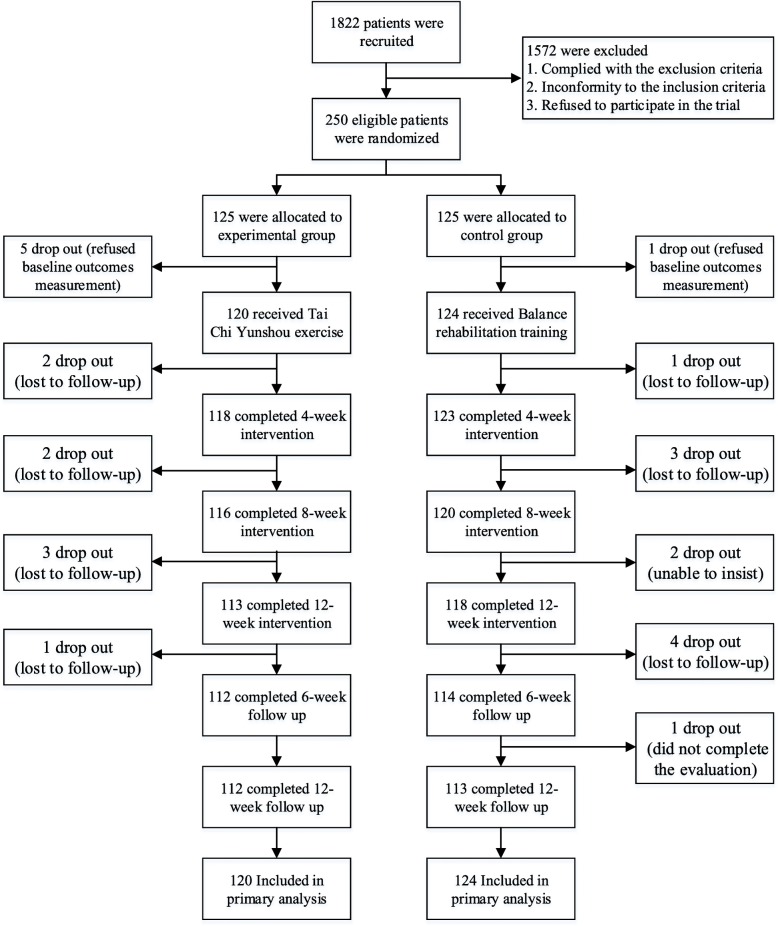
Participant flow through the study reported in a CONSORT diagram

Participant flow was showed as Fig. 1. Five participants in the TC group and one in balance rehabilitation group were dropped out before baseline assessments Therefore, they were not included in the ITT analysis. During the entire study period, eight participants in the TC group dropped out and eleven in the control group dropped out, and 112 (89.6%) participants in the TC group and 113 (90.4%) in the control group completed the study. The Fisher’s exact text indicated that there was no significant difference in the dropout rates between groups (χ^2^ = 0.413, *P* = 0.521, Table 1). However, not all individuals adhered to the complete training plan. The reasons given for absence including visits to physicians, health examinations, family dinners or other commitments that conflicted with training time. Finally, of the total 112 participants in the TC group, most participants had an attendance rate greater than 75% (48 participants attended more than 85% of the time and 59 participants, 76–85%), and a few subjects had an attendance rate of 75% or less (5 participants).

**Table 1:** Comparison of missing cases between two groups

	Tai Chi (*n* = 120)	Control(*n* = 124)	χ^2^ value	*P* value
End of research (missing data/ complete data) (n)	8/112	11/113	0.413	0.521

A total of 244 participants were included in final analysis; 74.6% were male, the average age was 60.9 y in the TC group and 60.1 y in the control group, and 71.7% had an ischemic stroke. The average length of the disease course was 14.5 months in the TC group and 14.3 months in the control group. There were no significant differences in baseline demographic characteristics between groups (including sex, age, education, duration of disease, MMSE, BBS, SLST, SF-36), and there were significant differences in FMA, MEFS, TUGT, MBI, and BDI (*P* < 0.05) between groups (Table 2).

**Table 2:** Baseline demographic characteristics for the 2 groups

Parameters	Tai Chi (*n* = 120)	Control (*n* = 124)	Group Difference	
			T (Z/F,χ^2^) value	*P* value
Age, mean ± SD (years)	60.9 ± 8.7	60.1 ± 8.6	*T* = 0.782	0.435^b^
Sex (male, %)	83 (69.2%)	99 (79.8%)	χ^2^ = 3.665	0.056^a^
Education (years)	9.6 ± 3.9	9.6 ± 3.8	Z = −0.226	0.821^c^
Duration of disease (month)	14.5 ± 18.1	14.3 ± 22.1	Z = −0.046	0.963^c^
Type of stroke (Ischemic, %)	89 (74.2%)	86 (69.4%)	χ^2^ = 0.696	0.404^a^
Hemiplegia side			χ^2^ = 5.124	0.163^a^
Left, %	67 (55.9%)	64 (51.6%)		
Right,%	36 (30.0%)	51 (41.1%)		
Bilateral,%	3 (2.5%)	2 (1.6%)		
No hemiplegia,%	14 (11.6%)	7 (5.7%)		
Handedness (L, %)	119 (99.2%)	122 (98.4%)	χ^2^ = 0.305	0.581^a^
MMSE(score range: 0–30)	27.2 ± 1.6	27.3 ± 1.6	Z = −0.599	0.549^c^
BBS(score range: 0–56)	33.5 ± 13.7	32.81 ± 12.0	Z = −0.686	0.493^c^
SLST (s)				
healthy side	12.7 ± 20.5	8.2 ± 12.7	Z = −1.566	0.117^c^
affected side	3.0 ± 6.1	2.6 ± 4.8	Z = −0.878	0.380^c^
FMA(score range: 0–100)	53.6 ± 25.3	47.0 ± 24.0	*T* = 2.082	0.038^b^
TUGT (s)	55.1 ± 73.9	42.0 ± 28.0	Z = −1.576	0.115^c^
MBI(score range: 0–100)	76.0 ± 20.7	71.0 ± 19.1	Z = −2.320	0.020^c^
MEFS(score rang:1–140)	77. 5 ± 35.6	58.7 ± 34.8	Z = 4.185	<0.001^c^
SF-36				
PCS(score range: 0–400)	158.4 ± 77.3	152.0 ± 80.6	Z = −0.716	0.474^c^
MCS(score range: 0–400)	167.3 ± 91.3	177.9 ± 77.0	*T* = −0.977	0.330^b^
BDI(score range:0–63)	18.3 ± 11.8	14.4 ± 10.2	*T* = 2.847	0.005^b^

All continuous variables are described using means and standard deviations

^a^performaned by Pearson χ^2^ test; ^b^performaned by the t-test; ^c^performaned by Mann-Whitney test

Abbreviations: *MMSE* the Mini-Mental State Examination, *BBS* Berg Balance Scale, *SLST* Single leg stance test, *TUGT* Timed-Up-and-Go test, *FMA* Fugl-Meyer, *MBI* Modified Barthel Index, *MFES* Modified Falls Efficacy Scale, *SF-36* the Mos Study 36-Item Short-Form Health Survey, *PCS* physical component summary, *MCS* mental component summary, *BDI* Beck Depression Inventory

**Fig. 2:**
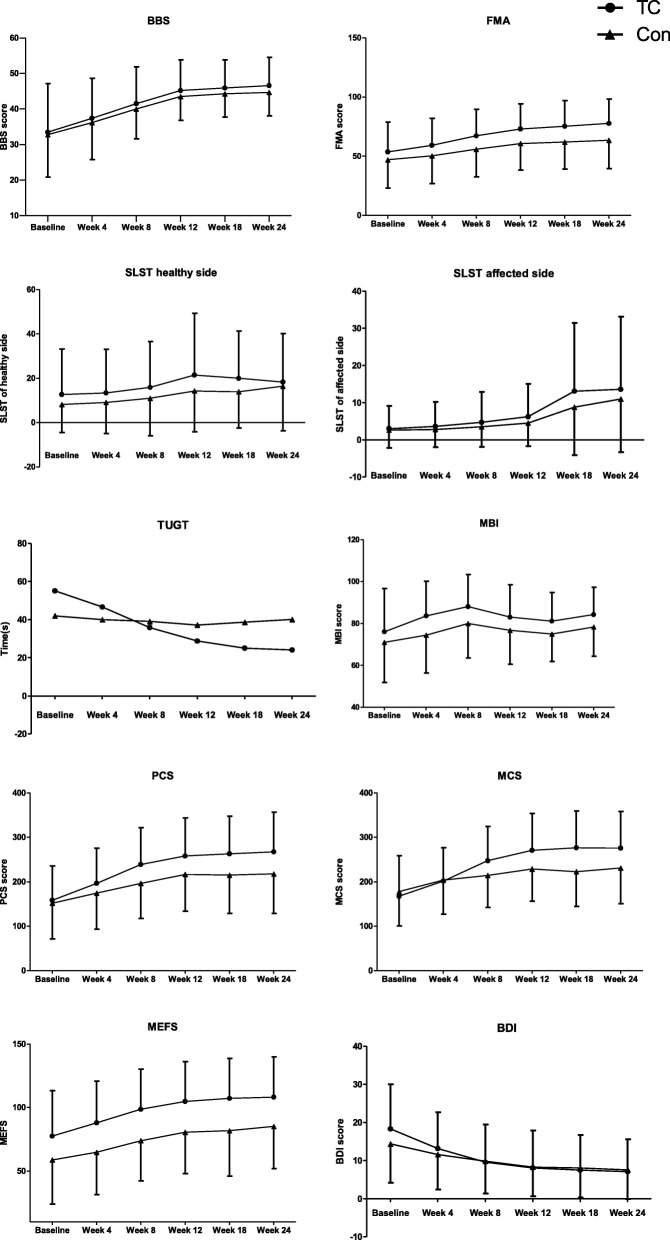
Comparison between Tai Chi Yunshou and control ggroups in the outcomes over time

Both Tai Chi Yunshou exercise and balance rehabilitation training had showed improvement by end of 12 weeks intervention over times (Fig. 2). The changes in outcomes for the Tai Chi Yunshou and balance rehabilitation group from baseline to the end of the 12 weeks intervention and to the end of the 12 weeks follow-up was showed in Table 3. For the primary outcome, BBS, the similar impovement was observed between groups on the change of week 12 to baseline and change of week 24 to baseline (*P* = 0.915, *P* = 0.715, respectively), and representing no significant difference after adjustment for baseline using a general linear model (*P* = 0.531, Table 4). For another balance parameter, SLST, the similar improvement was also observed between Tai Chi Yunshou and balance rehabilitation training after adjustment for baseline using a general linear model (*P* = 0.102, *P* = 0.221, respectively, Table 4). Of the parameter for quality of life, the similar effect of Tai Chi Yunshou and balance rehabilitation training was observed after adjustment for baseline using a general linear model (PCS:*P* = 0.063 and MCS:*P* = 0.052, respectively, Table 4).

**Table 3:** Outcomes on physical and psychological health (Mean ± SD/ Median (Inter-quartile range)

Outcomes	Groups	Baseline	End of 12 week intervention period	Difference of 12 week intervention to Baseline	T/Z Value	*P* Value	End of 12 week follow-up period	Difference of 12 week follow-up to baseline	T/Z Value	*P* Value
BBS	TC	37 (22.25~44)	47 (41~51)	8 (4~16)	−0.107	0.915	48 (43~52)	8 (4.3~20.3)	−0.336	0.715
	Con	34 (25~41)	43.5 ± 6.7	9 (5~14)			45 (42~49)	10 (5~16)		
SLST (healthy)	TC	5 (1~14.4)	12 (5.5~23.8)	4.0 (1~11.0)	−2.129	0.033	10.2 (5~25)	3(−2~15.8)	−0.886	0.387
	Con	3 (0~10)	8.8 (3~15)	2.5 (0~6.3)			12 (4.1~20.3)	3 (0~11.9)		
SLST (affected)	TC	0.265 (0~3)	3 (1.5~6.8)	2 (0~4)	−2.136	0.015	7.3 (3.1~14.8)	5 (1.3~11)	−1.255	0.210
	Con	0 (0~3)	2.8 (0~5.8)	0 (0~3)			7 (3~13)	3.1 (1~9)		
FMA	TC	50 (31.3~77)	78.5 (57~90)	13.1 (5.2~30.5)	−1.524	0.128	84.5 (62~94)	17 (6~33)	−1.911	0.056
	Con	45 (25~63)	59 (40~78.8)	11 (7~17.8)			66 (43.3~83.8)	14 (7.3~21.8)		
TUGT	TC	27.8 (16~54.9)	23.5 (14.7~34.1)	−3.9(−13.7~ − 0.7)	−0.879	0.370	18.8 (13.8~32.1)	−4.1(−15.9~1.2)	−1.625	0.104
	Con	42.0 ± 28.0	33.0 (18.8~50.3)	−5(−12.9~0)			34.33 (21.0~53.6)	−3.7(−13.0–2.6)		
MBI	TC	79 (63.3~95)	85 (73~98.8)	4.5(−4.0~15.8)	−0.235	0.814	85 (75.5~96.1)	4.0(−3.0~16.8)	−0.011	0.991
	Con	69 (61~89.75)	76.7 ± 16.1	5.6 + 16.1			79 (68.1~85)	7.3 + 15.2		
MEFS	TC	77. 5 ± 35.6	114 (83.3~131.8)	17.5 (8~42.9)	−0.508	0.611	114.5 (83~139.8)	19.5 (6.3~55.3)	−0.208	0.370
	Con	53 (29.3~83.6)	80.5 ± 32.5	19.5 (10~29)			85.1 ± 33.2	22 (10~38.7)		
PCS	TC	158.4 ± 77.3	276 (190.5~328.8)	78.9 (16.3~169.5)	−2.692	0.007	266 (207.4~350)	81.5 (15~185)	−2.847	0.004
	Con	132 (95.8~208.8)	216.8 ± 82.8	50 (15.3~108.0)			217.8 ± 88.5	45 (10~113.1)		
MCS	TC	167.3 ± 91.3	295 (192.1~335.8)	75 (18~182)	−3.753	< 0.001	293.5 (202~353.2)	86.5 (12~184.5)	−3.361	0.001
	Con	177.9 ± 77.0	236.5 (175~290.8)	35.7 (4.8~85)			241.5 (171.3~290.8)	35.3 (0~104.3)		
BDI	TC	16 (9~27)	4.31 (0~10.8)	−7(−16.8~ − 0.5)	−2.109	0.035	4 (0.93~10)	−8.5(−18~ − 0.2)	−2.136	0.033
	Con	13 (6~20)	6 (2.3~12)	−5(− 10.8~0)			5 (1.9~12.8)	−5(−12~ − 1)

**Table 4:** The effects of the Tai Chi Yunshou on the outcomes

Outcomes (score range)	Scourses	Sum of Squares	*F* Value	*P* Value	effect size Value
BBS(0–56)	Time	4060.833	38.407	< 0.001	0.139
	Group	63.321	0.394	0.531	0.002
	Group×Time	120.37	1.138	0.325	0.005
SLST(s) (healthy side)	Time	8146.268	12.521	< 0.001	0.050
	Group	670.850	2.689	0.102	0.011
	Group×Time	1103.528	11.696	0.168	0.007
SLST(s) (affected side)	Time	219.337	0.641	0.486	0.003
	Group	79.723	1.507	0.221	0.006
	Group×Time	193.981	0.567	0.521	0.002
FMA(0–100)	Time	14,548.498	41.520	< 0.001	0.148
	Group	9796.797	5.332	0.022	0.022
	Group×Time	2613.783	7.460	< 0.001	0.030
TUGT(s)	Time	746.657	0.330	0.645	0.001
	Group	22.076	0.042	0.839	< 0.001
	Group×Time	21,802.737	9.627	0.001	0.020
MBI(0–100)	Time	4212.047	15.487	< 0.001	0.061
	Group	963.320	1.511	0.220	0.006
	Group×Time	629.350	2.314	0.080	0.010
MEFS(1–140)	Time	21,175.317	21.490	< 0.001	0.082
	Group	14,144.527	30.760	< 0.001	0.114
	Group×Time	1662.543	632.574	0.175	0.007
SF-36					
PCS(0–400)	Time	69,737.418	7.667	< 0.001	0.031
	Group	11,316.527	3.501	0.063	0.014
	Group×Time	42,651.626	4.689	0.005	0.019
MCS(0–400)	Time	19,770.236	1.899	0.136	0.008
	Group	10,608.506	3.825	0.052	0.016
	Group×Time	94,181.540	9.047	< 0.001	0.037
BDI(0–63)	Time	4374.532	37.971	< 0.001	0.137
	Group	1386.080	4.490	0.035	0.018
	Group×Time	1246.265	10.818	< 0.001	0.043

Abbreviations: Berg Balance Scale *BBS*, Fugl-Meyer Assessment *FMA*, Single Leg Stance Test *SLST*, Timed-Up-and-Go-Test *TUGT*, Modified Barthel Index *MBI*, Modified Falls Efficacy Scale *MFES*, the Mos Study 36-Item Short-Form Health Survey *SF-36*, physical component summary *PCS*, mental component summary *MCS*, Beck Depression Inventory *BDI*

Futhermore, the Tai Chi Yunshou had more positive effects on the motor function, fear of falling and depression than balance rehabilitation training. For the parameter of motor function, the difference between two groups in the FMA was observed after adjustment for baseline using a general linear model (*P* = 0.022). The difference between two groups was also observed on fear of falling after adjustment for baseline using a general linear model (*P* < 0.001). The significant difference between groups was observed on depression parameter (*P* = 0.035).

### Safety

There was no adverse events related to Tai Chi Yunshou occurred in this trial.

## Discussion

In this cluster randomized controlled trial, we defined the balance rehabilitaion training as positive control, which is known as standard method for the balance rehabilitation of the stroke surivors according the guideline [44]. Compared Tai Chi Yunshou exercise with positive control on the physical and mental health of stroke survivors, we found the similar effectiveness of two intervention methods for parameters of balance ability (BBS, SLST and TUGT), activities of daily living (MBI), quality of life (PCS and MCS) over a 12-week intervention period. Furthermore, the improvement of motor function (FMA score), fear of falling (MFES), depression (BDI) in the Tai Chi Yunshou exercise is more effective than balance rehabilitation over a 12-week intervention period, which indicated that Tai Chi Yunshou has benefits on balance function, motor function, fear of falling and quality of life for community-based stroke survivors. The was no adverse events reported during the intervention period, which suggested that Tai Chi Yunshou exercise is safe for this population.

Tai Chi Chuan is a traditional Chinese mind-body exercise. Participants in Tai Chi Chuan were asked to shift their center of gravity slowly and at a constant speed between the two legs, revolving around the waist, while at the same time keeping the body upright, the shoulders and head in an upright posture and relaxed, and the spine comfortably aligned. Therefore, this type exercise program is expected to improve proprioceptive awareness and kinesthetic sense [45–47]. Previous studies had demonstrated that Tai Chi Chuan exercise has benefits on balance ability and gait speed for stroke survivors [16, 18, 48]. The improvement of Tai Chi Chuan on psychological symptoms, such as depression has also been reported [49, 50]. However, the Tai Chi Chuan used in previous study were 6-form [27], 12-form [17] or 24-form [16] style which make the exercise challenging for the stroke survivors. The Tai Chi Yunshou (wave hands in the cloud) movement is the basic and “mother” form of all styles of Tai Chi Chuan. During Tai Chi Yunshou exercise, head-and-eye movements, the trunk and limbs movement are synchronous and frequent changes of head-and-body orientation are required. This may result in a better combination of the visual and vestibular systems for balance control. To this end, the single form (waves hands in cloud movement) of Tai Chi Chuan was used in this study to improvement the physical and mental health for stroke survivors.

In this study, balance ability was evaluated using BBS, SLST. The improvement of the balance ability between two group was not showed statistical significance. This is in agreement with previous research [26]. Hart et al. reported that both Tai Chi Chuan and general physical therapy can improve the balance ability (BBS, standing on one leg) and speed of walking and there was no significant difference between groups. The authors also suggested that Tai Chi Chuan can be used as an alternative and complementary therapy for the stroke with balance impairment [26]. In contrast, Au-Yeung indicated that a significant improvement in TUGT of stroke patients performed Tai Chi Chuan once a week for 12-week compared to control group [17]. In addition, Taylor-Piliae and Coull reported that stroke survivors exercised Tai Chi Chuan had balance and walking speed improved [16]. The explain maybe that those studies defined usual care as intervention of control group which can not improve the balance ability and mobility. For the quality of life parameter, both Tai Chi Yunshou exercise and balance rehabilitaion can improve the quality of life for the stroke survivors. This is consistent with previous research [15, 18], which demonstrated that 12-week Tai Chi practice could benefit the quality of life of stroke survivors. The reason for this finding may be that this group activity provides social support and participation, which improves or preserves the quality of life.

Furthermore, we demonstrated that the Tai Chi Yunshou exercise and balance rehabilitation training can effective in improving the motor function, fear of falling and depression. A previous study with post-stroke survivors enrolled demonstrated an improvement in motor function after a 4-week Tai Chi exercise program compared to rehabilitation training [51], which is in accordance with current study. As a moderate intensity aerobic exercise combining static and dynamic movements [30], Tai Chi Yunshou asks subjects to place the center of their body weight on two legs, which can enhance the muscular strength of the lower limbs [34]. This may be the reason why Tai Chi Yunshou improved the motor function of stroke survivors. Our result was also similar to a recent study conducted by Lin [52], who assessed the effectiveness of Tai Chi Chuan exercise on fall-related outcomes among frail adults compared with balance rehabilitation program. They found that Tai Chi Chuan was more effective in improving fear of falling than general physical therapy. The improvement in balance ability by Tai Chi Yunshou was not agree with changes in motor function as measured by FMA. This result elucidated that the effect of Tai Chi Yunshou exercise was specific to the task being trained, namely, balance ability by performing head, body with weight shifting. The Tai Chi Yunshou form do not stress turning. As a body-mind exercise, the meditative and relaxation training of Tai Chi has been shown to reduce anxiety and depression [53]. The reason for this finding may be that Tai Chi Yunshou emphasizes sustained attentional focus and mind-regulation during movement, which may impact cortisol and other stress-related pathways.

More than 85% of the subjects completed the experiment, and at least 95% of the subjects were included in the analysis. Only 20 cases fell off (a drop-out rate of 8%). No adverse events occurred. A series of measures were carried out to ensure the adherence and safety of the participants. First, our class sizes were small (maximum of five/group) so that the intervener could provide on-site guidance and supervision sufficiently. Second, CHCs near the subjects were selected as training venues to ensure compliance. Third, those who could not exercise for 1 h continuously could complete the session in sections, which were not less than 15 min each section. Fourth, subjects with severe upper limb dysfunction were allowed to perform the exercises by using the unaffected hand to aid the affected hand. Additionally, health education was provided to all individuals throughout the intervention period. Weekly telephone follow-ups and monthly family follow-ups were used to improve the return ratio. Another important reason we hypothesize for the small dropout rate is that Tai Chi Yunshou exercise is constructed as a group training, community-based program, in which individuals can receive encouragement including social support and knowledge acquisition [2].

## Conclusion

In conclusion, the present study demonstrated that both a 12-week Tai Chi Yunshou exercise and balance rehabilitation led to impoved balance ability and functional mobility, and both are suitable community-based programs that may benefit for stroke recovery and community reintegration. Our data suggested that a 12-week Tai Chi Yunshou intervention was more effective in improving motor function, fear of falling and quality of life than balance rehabilitation. The basic form of Tai Chi Chuan, waves hands in cloud movement, can be used as an alternative and complementary therapy for the community-based stroke survivors with balance impairment.

### Limitations

There are also some limitations in this study. First, although it was impossible to blind the participants and instructors in this study, the outcome evaluators and analysts were blinded to reduce bias. Second, participants were not asked to record their daily exercise, including the type, exercise time, frequency, etc., during the study, which could also be a confounding factor that affected the results. Additionally, there was no supervision during the follow-up period, which may have magnified the effects due to the self-initiated exercise of patients. In addition, the observed improvements in physical function among participants in this study may have occurred as a result of a learning effect, associated with repeated use of the study measures to assess changes after the 12-week intervention. We did not check the intensity of two interventions, which can effect the results.

## Abbreviations

ADL: Activities of daily living
BBS: Berg Balance Scale
BDI: Beck Depression Inventory
CHCs: Community health centers
FMA: Fugl-Meyer Assessment
ITT: Intention-to-treat
MBI: Modified Barthel Index
MFES: Modified Falls Efficacy Scale
SF-36: The Medical Outcomes Study 36-Item Short-Form Health Survey
SLST: Single Leg Stance Test
TUGT: Timed-Up-and-Go-Test
